# Place-based estimates of cigarette butt litter raise environmental justice concerns in the United States

**DOI:** 10.1371/journal.pone.0308930

**Published:** 2024-08-15

**Authors:** Alexander T. Lowe, Alexander Maki, Carla Figueroa, P. Dilip Venugopal

**Affiliations:** 1 Division of Nonclinical Science, Center for Tobacco Products, U.S. Food and Drug Administration, Silver Spring, MD, United States of America; 2 Division of Population Health Science, Center for Tobacco Products, U.S. Food and Drug Administration, Silver Spring, MD, United States of America; University of Catania, ITALY

## Abstract

Littering of cigarette butts is a major environmental challenge. In 2022, ~124 billion cigarette butts were littered in the United States. This litter may pose an environmental justice concern by disproportionately affecting human and environmental health in communities of color or communities of low socioeconomic status. However, the lack of data on the distribution and magnitude of cigarette butt littering prevents an environmental justice analysis and limits the ability to tackle this environmental challenge. We conducted an environmental justice assessment of tobacco product waste, specifically cigarette butts, through spatially-explicit, place-based estimates across the contiguous U.S. We built a bottom-up model by synthesizing census tract-level population and smoking prevalence, state-level cigarette consumption, and published littering data to assess the spatial pattern of cigarette consumption and littering, and its implications for environmental injustice in >71,600 U.S. census tracts. Further, we compared the model output to urbanicity (rural-urban commuting area) and Social-Environmental Risk (SER; CDC Environmental Justice Index). Cigarette butt density was not uniformly distributed across the U.S. and ranged from 0–45.5 butts/m^2^, with an area-weighted average of 0.019 ± 0.0005 butts/m^2^. Cigarette butt density was 96 times higher in metropolitan vs. rural areas. Cigarette butt density increased significantly with SER, with 5.6 times more littered cigarette butts, and a steeper response to population density, in census tracts with the highest SER vs. the lowest SER. These results demonstrate the relative influences of location, smoking prevalence, and population density, and show that cigarette butt littering is a potential environmental justice concern in the U.S. This study provides information that may help devise targeted strategies to reduce cigarette butt pollution and prevent disproportionate impacts. The spatial data layer with place-based cigarette consumption and butt density is a tool that can support municipal, state, and federal level policy work and future studies on associations among cigarette butt pollution and environmental health outcomes.

## Introduction

Environmental justice addresses the disproportionate and adverse effects resulting from the intersection of local demographics, socioeconomic status, health, and environmental policy and quality [1, 2]. Historically marginalized communities in the United States, including communities of color and communities of low socioeconomic status (SES), as defined by factors such as access to housing, employment, income, education, and healthcare [3], are disproportionately affected by exposure to environmental hazards. Individuals in these communities are more likely to be exposed to air pollution [4, 5], water pollution [6–9], toxic and chemical waste [10, 11], and other environmental hazards [12, 13]. The disproportionate exposure to these environmental stressors results in increased rates of asthma, cancer, cardiovascular disease, diabetes, and chemical poisoning [2, 14–16]. Individuals from marginalized communities also tend to face a higher risk for poor nutrition, lower education, inadequate housing, and higher health-risk behaviors because of the interacting social and economic vulnerabilities [4, 17, 18]. Understanding the cumulative place-based factors affecting public and environmental health helps to identify disparities and may suggest ways to advance environmental justice and health equity [19, 20].

Tobacco products contribute to health disparities in marginalized communities through numerous pathways [21, 22]. Tobacco product marketing targets people of color and communities of low SES [23, 24] and tobacco retailers are more likely to be located in these communities [25–27]. Individuals who are of low SES report greater use of cigarettes, have lower odds of quitting tobacco products [28, 29], and are more likely to be exposed to second-hand tobacco smoke in general [30–32]. Furthermore, individuals in marginalized communities are less likely to have access to cessation programs and are more likely to experience negative health outcomes related to tobacco use, including higher rates of cancer and chronic obstructive pulmonary disease [24, 33, 34]. Research on tobacco-related health disparities has largely focused on health outcomes associated with marketing and use of tobacco products. However, it is still unknown if the disposal of tobacco products poses similar disproportionate environmental health burdens for marginalized communities.

Littered cigarette butts are one of the biggest environmental health hazards related to the disposal phase of tobacco products. In 2022, Americans consumed approximately 191 billion cigarettes, of which 99.8% are filtered cigarettes [28, 35, 36]. An estimated 65% of these cigarettes are littered [37], resulting in nearly 124 billion cigarette butts, or 74 million pounds of cigarette litter (assuming 0.27 g/butt [38]), entering the environment. Cigarette butts pose a ubiquitous environmental hazard as they are among the most common littered item found in beach cleanups [39–41], urban areas [42], and along roadways [43, 44]. Efforts to quantify and map cigarette butt litter on beaches and urban areas have shown the pervasiveness of cigarette butt littering and its contribution to local pollution [39, 45, 46] and the cost of cleanup [47].

Littered cigarette butts can directly impact people and pose risks to pets and other wildlife through consumption and poisoning exposures, which primarily affect young children [48–50]. Cigarette butts can release harmful chemicals to the air [51, 52], but a larger concern is the release of chemicals and microplastics into water and soil [53]. Cigarette butt leachate is composed of numerous toxic compounds, including contaminants of concern such as phthalates, in concentrations that can affect aquatic organisms and water quality [54]. In addition, cigarette filters contain cellulose acetate, a plastic material with poor biodegradability that can take more than 10 years to degrade and contributes to cigarette butt litter accumulation and the introduction of microplastics into the environment [55–57]. The ubiquity and longevity of cigarette litter, the concentrations of toxic chemicals in cigarette litter, and the associated contribution to microplastic pollution pose a risk to human and environmental health [54, 58, 59].

Studies have estimated cigarette butt litter to inform global economic costs [60], estimate clean-up costs in U.S. cities [47], evaluate environmental benefits of policies such as a menthol ban [61], and map cigarette butt litter within a single city [62, 63]. These studies advance the methodological aspects of estimating cigarette butt litter including considerations of use prevalence and population information, and geographic information systems (GIS) approaches. They also inform efforts to address tobacco product waste. Cigarette butt litter is a putative environmental justice issue because littering tends to be higher near vendors [63], and tobacco vendors tend to be disproportionately concentrated in marginalized communities [25, 27, 64]. Any effort to understand environmental justice issues related to disposal of tobacco products needs to quantify tobacco product litter in the United States and map its distribution. The total amount and nation-wide distribution of cigarette butt litter remains poorly understood and placed-based, localized estimates of cigarette butt litter for the entire U.S. are currently not available.

We conducted a national-scale environmental justice assessment examining cigarette butt litter in relation to existing environmental burdens and social vulnerabilities of communities. We first constructed a bottom-up, place-based model of cigarette consumption and littering at the census tract scale using publicly available data for the contiguous U. S. We then compared the estimated cigarette butt litter density to Social and Environmental Risk (SER) from the Environmental Justice Index (EJI) [3]. Such a place-based assessment is useful to identify if cigarette butts add to cumulative, disproportionate environmental health burdens for communities already facing social vulnerabilities. Protecting the health of historically marginalized communities on the frontlines of pollution and other environmental hazards is the mission of federal efforts per Executive Orders 12898 and 14096 on Environmental Justice [65, 66], the U.S. Health and Human Services–Office of Environmental Justice [67], and the U.S. Food & Drug Administration’s One Health Initiative that recognizes the interconnectedness of human, animal, and environmental health [68]. An improved understanding of the quantity and distribution of cigarette butt litter can inform FDA’s environmental assessments of new tobacco products under the National Environmental Policy Act (NEPA) mandate [69] by defining affected areas and investigating disposal-related impacts and environmental justice concerns [70]. Data from this paper may be used by regulators and policymakers and inform education strategies.

## Methods

### Modeling littered cigarette butts

The bottom-up model of cigarette use and littering across the contiguous U.S. was constructed using publicly available data and published values. The model integrated social factors including prevalence of cigarette use, population size, the average number of cigarettes used per individual, and estimated cigarette butt littering.

### Number of individuals who smoke

Census tract-level estimates of smoking prevalence were obtained from the Centers for Disease Control and Prevention (CDC) PLACES dataset [71]. PLACES data are based on the 2020 CDC Behavioral Risk Factor Surveillance System survey (BRFSS) [72]. In the BRFSS, individuals who currently smoke are defined as adults aged 18 years or older who reported having smoked ≥100 cigarettes in their lifetime and currently smoke every day or some days. PLACES created small area estimation measures for each census tract from the BRFSS data using a multilevel statistical model. The total number of individuals who smoke per census tract was calculated by multiplying the smoking prevalence by the 2020 population in that tract, both as reported in the PLACES data.

### Cigarette consumption

It is optional for states to collect BRFSS data on the number of cigarettes smoked per individual. Given the limited BRFSS data, we instead used the 2018–2019 Tobacco Use Supplement to the Current Population Survey (TUS-CPS) state-level estimates of the number of cigarettes consumed per individual per day (CPD) [73]. The TUS-CPS is a National Cancer Institute-sponsored survey of self-reported tobacco use that has been administered as part of the U.S. Census Bureau’s Current Population Survey. Data on CPD are available by state and the District of Columbia. The range of mean state-level CPD (5.82–14.97) is lower than other estimates from the literature (Table 1), but the spatial representation and standardized methodology make this source the most appropriate for the current effort. Each census tract estimate of the number of individuals who smoke was multiplied by the relevant state-level CPD estimate, and then multiplied by 365 days, to calculate the total number of cigarettes used in each census tract per year.

**Table 1:** Estimates of cigarettes used per day and proportion of cigarettes littered.

Measure	Source	Location	Type of study	Sample Size	Estimate	Description
Cigarettes per day	Shiffman, 2009 [74]	United States	Self-reported	232	~24	
	Hughes et al., 2010 [75]	U.S.	Self-reported Survey	23393	16.4	
	Benowitz et al., 2011 [76]	U.S.	Lab study	128	17.8	
	U. S. Center for Disease Control and Prevention, 2018 [77]	U.S.	Self-reported Survey	Weighted to U.S. adult population	14	
	Klemperer et al., 2019 [78]	U.S.	Self-reported Survey	132	20	
	Nighbor et al., 2021 [79]	U.S.	Secondary analysis of randomized trial	68	~21	
	2018–2019 TUS-CPS [73]	U.S.	Self-reported Survey	Weighted to U.S. adult population	11.1	Unweighted mean of states; state level data used in model.
Proportion of cigarettes littered	Morgan et al., 2022 [80]	U.S.	Survey	719	62.2%	Percent of individuals reporting littering.
	Nitschke et al., 2023 [81]	Sweden	Behavioral observation	597 smoking events	80.0%	Percent of individuals who litter.
	Patel et al., 2013 [44]	New Zealand	Behavioral observation	219 smoking events	76.7%	219 cigarette discarding events in central business district of Wellington, New Zealand.
	Rath et al., 2012 [82]	U.S.	Self-reported survey	2,000	74.1%	Percent of individuals reporting littering.
	Schultz et al., 2013 [37]	U.S.	Behavioral observation	767 smoking events	65%	Urban, suburban, and rural settings in each of 10 U.S. states; 44 locations total.
	Sibley et al., 2003 [83]	NZ	Behavioral observation	278 smoking events	98.7%	Percent of individuals who litter.
	Wilson et al., 2014 [46]	NZ	Behavioral observation	112 smoking events	84%	Bus stops in two New Zealand cities

Summary of estimates of cigarettes consumed per day and littering rates from published studies for comparison to the values used in the model.

### Number of littered butts

The limited number of studies quantifying littering rates of cigarette butts generally use survey methods to estimate the proportion of individuals who litter or observational studies to count the number of cigarettes butts that are littered after use (Table 1). Survey estimates of the percent of individuals who litter their cigarettes ranged from ~60–100%, but these estimates are difficult to translate into littered cigarette butts or generalize to the U.S. population. These survey estimates do not provide information on the percent of an individual’s cigarettes that are littered. Observational estimates of the percent of cigarettes that are littered after use ranged from 65–84% and can be used directly to estimate littered cigarettes (Table 1). These observational estimates are made in public settings and may not reflect the full range of an individual’s littering behavior. However, no spatially-explicit or demographic-specific estimates of littering behavior, either from surveys or observations, are available. For this model, we used the estimate of 65% of butts being littered [37]. Schultz et al. (2013) observed cigarette butt littering rates across a range of locations (e.g., city centers, gas stations, rest stops), population densities (i.e., urban, suburban, and rural cities), and geographic regions (i.e., states) in the U.S. that are most representative of the current study area. We viewed the Schultz et al. (2013) study as having strengths relevant to the present purposes as compared to studies generating self-reported rates of cigarette butt littering or collecting behavioral observations from other countries (see references in Table 1). This estimate has been used in at least one other study estimating national littering rates [61]. The total estimate of used cigarettes in each census tract per year was multiplied by 65% to estimate the number of littered cigarette butts per year in each census tract, which were then converted to cigarette butt density by dividing by the area of the census tract. We examined spatial autocorrelation in area-weighted mean cigarette butt density per census tract using Global Moran’s *I* test statistic with a first-order queen contiguity spatial weight matrix with equal weights, defining the neighborhood structure of each census tract. We tested the statistical significance of Global Moran’s *I* value using a permutation test (Monte-Carlo simulation; 999 permutations) and generated a spatial lag variable, all using *spdep* package [84, 85] in R program [86]. The map of cigarette butt density was created in QGIS [87] with spatial data on census tract boundaries available freely through the United States Census Bureau [88].

### Cigarette butt litter and rural–urban populations

Area-weighted mean cigarette butt density was compared among categories of urbanization using the United States Department of Agriculture Rural-Urban Commuting Area data (RUCA) [89]. RUCA classifies each census tract as: “Metropolitan” for census tracts in which >30% of the population is in an urban area, “Micropolitan” for census tracts in urban clusters with a population of 10,000–49,999 individuals, “Small Town” for census tracts in an urban cluster with 2,500 to 9,999 individuals, three “Commuting” classifications for census tracts outside of urban areas and urban clusters but with >10% of the population commuting to Metropolitan, Micropolitan, or Small Town, respectively, and “Rural” for census tract with primary commuting flow not going to an urban area or cluster. RUCA classification was based on the 2010 census. Census tract total cigarette butt litter was compared among RUCA categories using a quasi-poisson generalized linear model (GLM) with census tract area as an offset. The census tract area offset standardized cigarette butt counts to area (m^2^), such that the model estimates provide area-weighted means for each RUCA category. Post-hoc comparisons among RUCA categories were run using Dunnett’s test with RUCA–Rural category as the control group as smoking prevalence is higher in rural areas [90].

### Cigarette butt litter and environmental justice

Potential cumulative effects of littered cigarette butts on environmental justice were investigated by testing associations between modeled cigarette butt litter and local social and environmental risk. We used the SER module of the EJI [3]. The SER module provides a combined percentile ranking (range 0.0–1.0) for each U.S. census tract that represents the cumulative environmental burdens and social vulnerabilities. A percentile ranking represents the proportion of tracts that are equal to or lower than the tract of interest in environmental burden and social vulnerability. Higher SER percentile rank indicates higher combined vulnerabilities [3, 91]. This index is appropriate for secondary analyses with PLACES-based model output as the percentile ranking of SER variables and exclusion of small-area estimates of health outcomes prevent the introduction of auto-correlations [92]. Post-hoc comparisons among SER quintiles were run using Dunnett’s test with SER 0.0–0.2 quintile (lowest risk) as the control group. The incidence rate ratios and their 95% confidence intervals were calculated for all the GLMs and are reported. Further, cigarette butt count was compared among quintiles of SER, population, and the interaction between SER and population with a quasi-poisson GLM with log link function and with census tract area as an offset. Statistical analyses were conducted in R [86] with the ‘stats’ package version 4.1.1. Area-weighted standard errors of the mean were calculated in R with the ‘wtd.var’ function in the ‘Hmisc’ package version 5.0–1. Model estimates were extracted with the package ‘sjPlot’ [93] and Dunnett tests were performed with package ‘multcomp’ [94]. Data are available in the supplementary file (S1 Data).

### Model evaluation

Modeling cigarette butt density at the census tract level required collating data from multiple sources that vary in uncertainty. It would have been ideal to derive all behavioral estimates (i.e., smoking prevalence, number of cigarettes consumed, and number of littered cigarette butts) from the same source. However, there is no single source that includes all three types of estimates. Multiple methods were used to contextualize this uncertainty. First, the propagated error of the total number of littered cigarettes and area-weighted cigarette butt density was calculated from error of population and smoking prevalence estimates. Second, the modeled total number of consumed cigarettes was compared to the number of cigarettes sold in the U.S. in 2018–2020, as estimated from federal excise tax data, to assess whether the bottom-up calculation resulted in a similar total. The U.S. Alcohol and Tobacco Tax and Trade Bureau (TTB) compiles data on the number of cigarettes sold each year as a proxy for use [35]. The TTB data collection methods are independent of the methods used by the data sources in the model and thus provide a reasonable comparison point at the national scale. These data were not used to construct estimates of littering because they are not available at state or census tract scales. Third, we compared BRFSS smoking prevalence estimates to TTB sales data as a proxy for use from 1995 to 2021 [35, 95]. A correlation over time between these independently collected measures of smoking behavior in the U.S. would provide evidence that the BRFSS smoking prevalence metric is a good predictor of overall cigarette consumption in the U.S., and not just the number of people who smoke. The temporal trends in total cigarette consumption also provide valuable context for the model results that represent a single year of littering of a product that can persist and accumulate in the environment for over a decade.

Finally, to assess uncertainty related to littering behavior, we created three additional scenarios with different estimates of cigarette butt littering rates to compare to the model estimate of 65%. We used an upper bound estimate of 85% of cigarettes littered [46], an estimate of 35% which is much lower than published observational study values (Table 1), and 10% as a lower bound. The 10% littering scenario was based on a recent estimate from the Population Assessment of Tobacco and Health Study wherein ~90% of adults who use manufactured cigarettes self-reported that they usually dispose of cigarette butts in a landfill [96]. As discussed above, self-reported rates of the percent of individuals who litter are challenging to translate into the number of littered cigarettes. The value of 10% littering in this model scenario was determined by assuming the approximately 10% of adults who did not dispose of their cigarettes in the landfill littered all their cigarettes. Estimates of 85%, 35%, and 10% littering rate provide a range of scenarios to compare the magnitude of cigarette butt litter. The estimated proportion of cigarettes that are littered after use came from one study [37]; the limited number of studies with similar designs prevents calculation of a standard error associated with this estimate.

## Results

**Fig 1:**
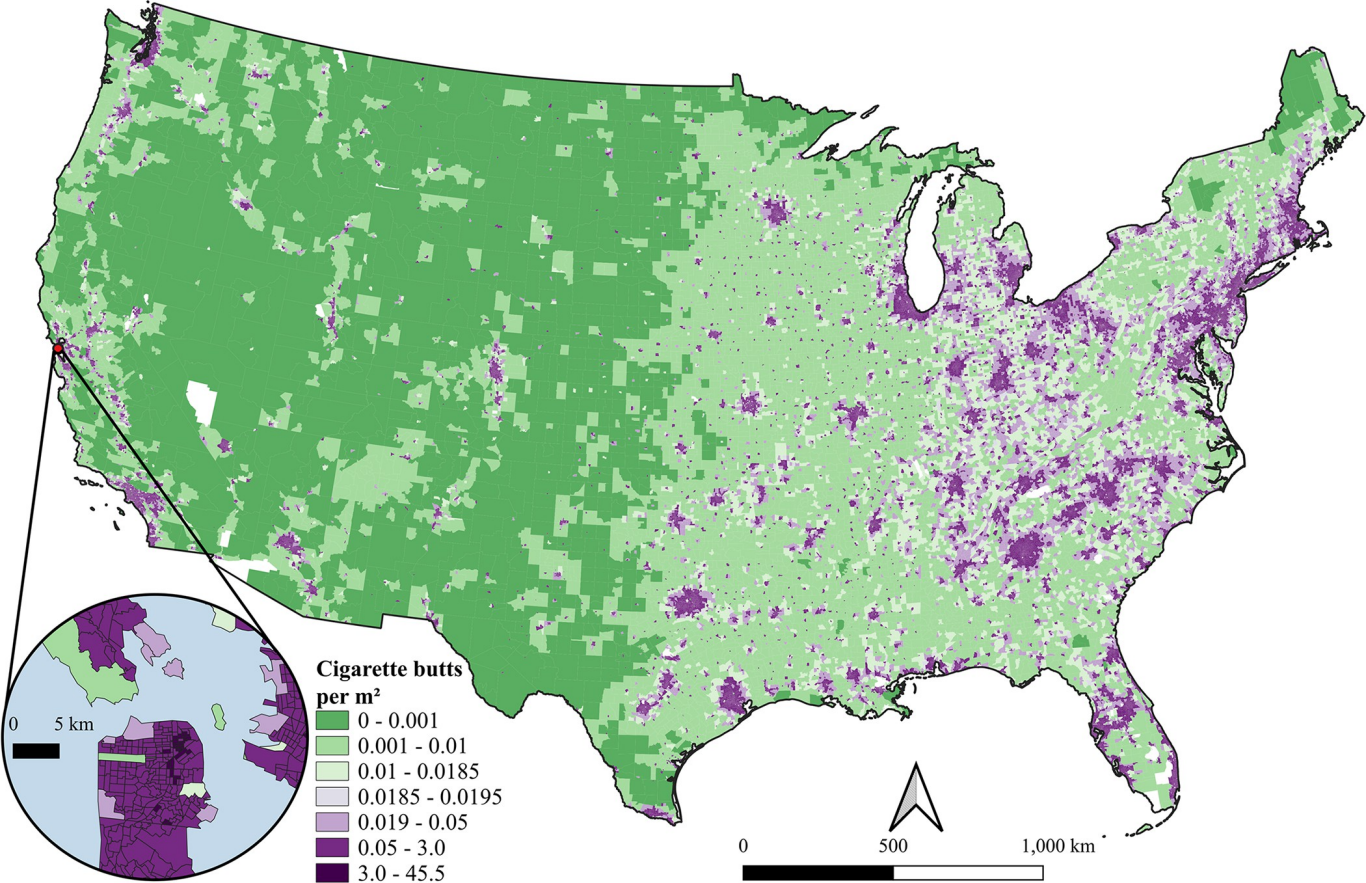
Littered cigarette butt density at the census tract scale across the contiguous United States. Color scale is centered on the mean +/- 1 SE in the light purple. White areas within the map are census tracts with missing data. Inset map shows census tract detail in the San Francisco area; water indicated in blue. The map was generated with spatial model output on census tract boundaries available to the public through the United States Census Bureau and the PLACES dataset.

The model estimated 145.77 billion cigarette butts were littered over one year. Census tract butt density ranged from 0–45.5 butts/m^2^, with an area-weighted average of 0.019 ± 0.0005 butts/m^2^ (Fig 1).

**Fig 2:**
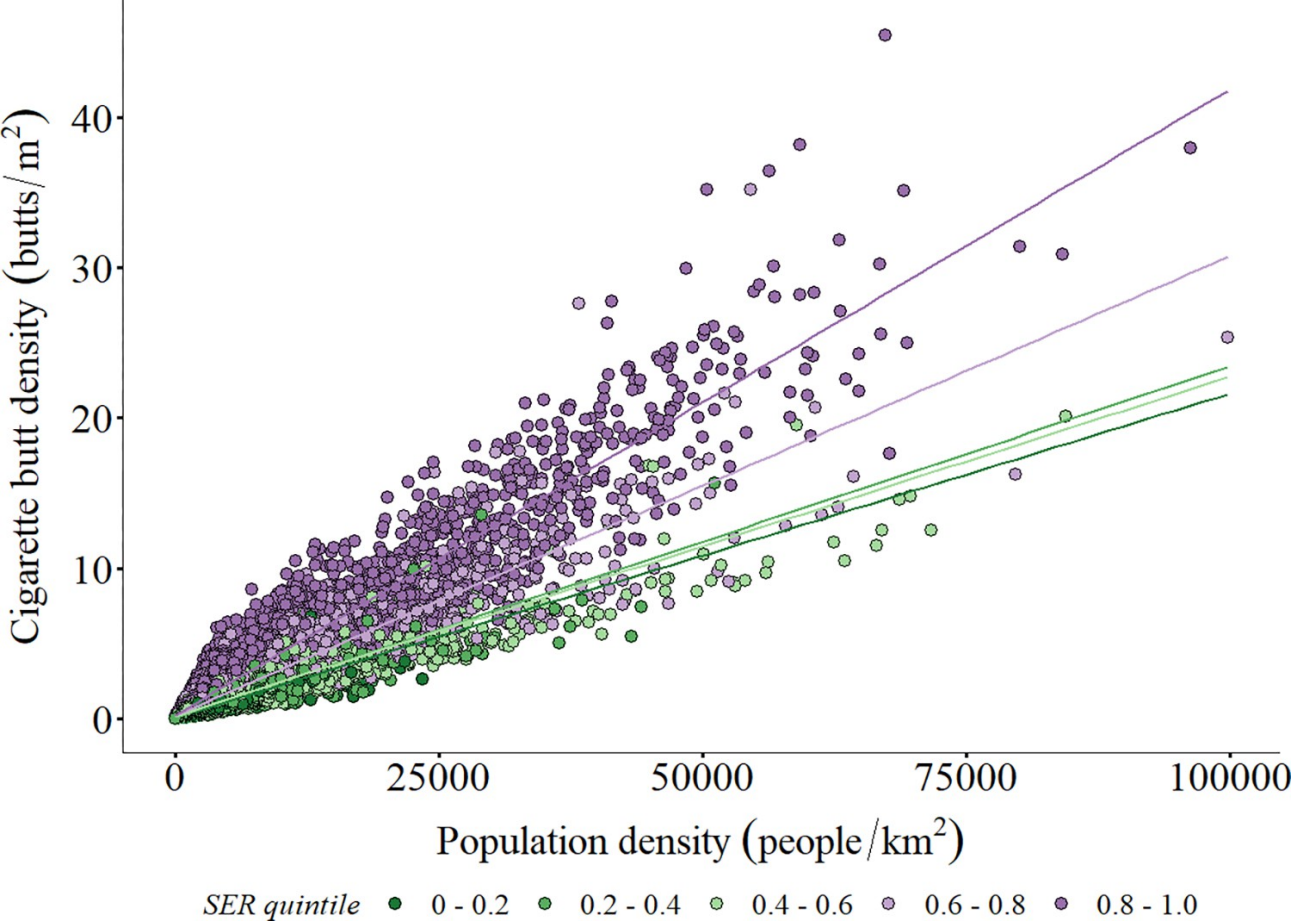
Comparison of cigarette butt density to population density (People/km^2^) among quintiles of Social-Environmental Risk. Each point represents a census tract. Social-Environmental Risk represents the percentile rank (from 0 to 1) of all census tracts. Lines are linear regression for data within a quintile.

The distribution of estimated cigarette butt litter was heterogeneous across the contiguous U.S. with significant spatial clustering. Global Moran’s *I* test statistic and the density plot of permutation outcomes (not shown) based on Monte-Carlo simulation of Moran’s *I* indicated significant global spatial autocorrelation in cigarette butt density in census tracts across the United States (Global Moran’s *I* = 0.75, *p* = 0.001). This can be seen in the higher cigarette butt densities in and around cities and in the eastern U.S. compared to the western U.S. (Fig 1). Area-weighted mean cigarette butt density varied significantly among categories of urbanization (quasi-Poisson GLM; Log-ratio *X*^*2*^ = 34,436.0, *df* = 6, *p* <0.0001; Table 2). Hotspots of littered cigarette butts were found in metropolitan areas (0.181 ± 0.002 butts/m^2^), with intermediate values found in micropolitan (0.029 ± 0.001 butts/m^2^) and small towns (0.010 ± 0.001 butts/m^2^), and lowest values in rural areas (0.002 ± 0.0001 butts/m^2^) (Figs 1 and 2). The average number of littered cigarette butts was 96.4 times higher in metropolitan areas compared to rural areas (Table 2).

**Table 2:** Area-weighted mean cigarette butt density among Rural-Urban Commuting Areas (RUCA).

Predictors	Butts per m^2^± SE	Incidence Rate Ratios (95% CI)	Wald χ^2^ / *Z* value	*p-value*
Rural (control)	0.002 ± 0.0001		34438	<0.001
Small Town-Commuting	0.004 ± 0.0002	1.83 (1.57 – 2.14)	7.62	<0.001
Small Town	0.010 ± 0.0006	5.51 (4.86 – 6.24)	26.70	<0.001
Micropolitan-Commuting	0.006 ± 0.0002	3.12 (2.76 – 3.53)	18.25	<0.001
Micropolitan	0.029 ± 0.0014	15.61 (14.01 – 17.41)	49.53	<0.001
Metropolitan-Commuting	0.011 ± 0.0019	5.63 (5.10 – 6.22)	33.89	<0.001
Metropolitan	0.181 ± 0.0018	96.36 (88.20 – 105.54)	99.79	<0.001

Area-weighted mean cigarette butt density (butts/m^2^), standard error (SE), and incidence rate ratio (IRR) among RUCA categories. Incidence rate ratios and intervals are back-transformed from the log scale. *Z value* estimates for post-hoc comparisons through Dunnett test, n = 71830.

Higher cigarette butt density was strongly correlated to increased SER (quasi-Poisson GLM; Log-ratio *X*^*2*^ = 1123.2, *df* = 4, *p* <0.0001; Fig 2; Table 3). Census tracts ranked in the top 20% of SER, representing those already facing the highest environmental hazards and social vulnerabilities, had on average 0.086 ± 0.003 butts/m^2^, 5.6 times higher than census tracts in the lowest quintile of risk, which averaged 0.015 ± 0.0005 butts/m^2^. The presence of large census tracts with low cigarette butt density resulted in area-weighted means that are much lower than the arithmetic mean. Cigarette butt density was positively correlated to census tract population density, with the highest cigarette butt density occurring in small, densely populated census tracts (quasi-poisson GLM; Log-ratio *X*^*2*^ = 1211.2, *df* = 1, *p* <0.0001; Figs 1 and 2). There was a significant interaction between SER percentile rank and population density (quasi-poisson GLM; Log-ratio *X*^*2*^ = 142.8, *df* = 4, *p* <0.0001). Cigarette butt density increased faster with population density among census tracts with higher SER than those with low SER (Fig 2).

**Table 3:** Area-weighted cigarette butt density among quintiles of Social-Environmental Risk (SER).

Predictors	Butts per m^2^ ± SE	Incidence Rate Ratios (95% CI)	Wald χ^2^ / Z value	*p-value*
SER: 0.0–0.2 (lowest risk) (control)	0.015 ± 0.001		1255.6	<0.001
SER: 0.2–0.4	0.012 ± 0.001	0.73 (0.64 – 0.83)	-4.85	<0.001
SER: 0.4–0.6	0.014 ± 0.001	0.88 (0.78 – 1.00)	-2.02	0.13
SER: 0.6–0.8	0.027 ± 0.001	1.79 (1.58 – 2.03)	9.15	<0.001
SER: 0.8–1.0 (highest risk)	0.086 ± 0.003	5.6 (4.95 – 6.33)	27.54	<0.001

Area-weighted mean cigarette butt density (butts/m^2^), standard error (SE), and incidence rate ratio (IRR) among quintiles of SER. Incidence rate ratios and intervals are back-transformed from the log scale. *Z value* estimates for post-hoc comparisons through Dunnett test, n = 71677.

### Model evaluation

**Fig 3:**
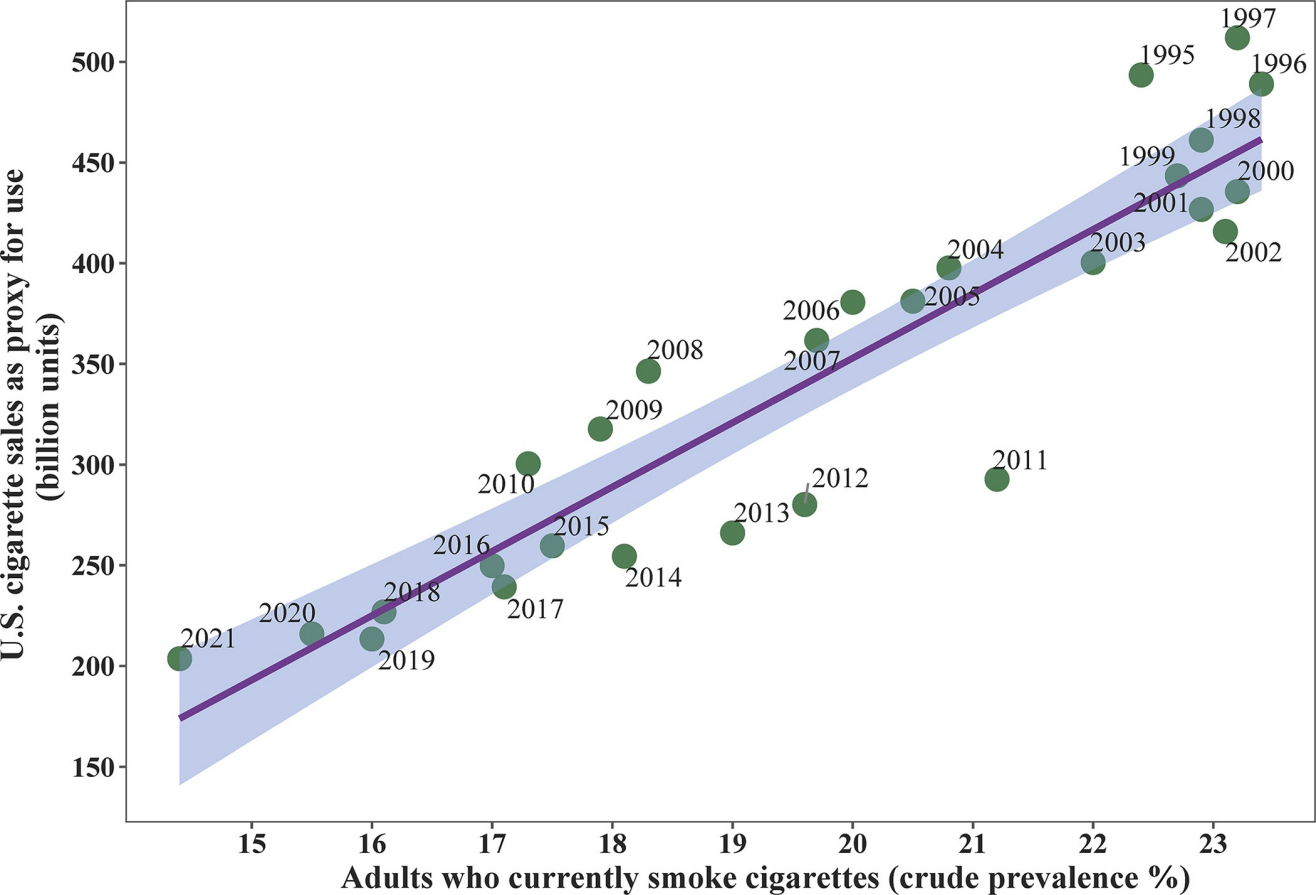
Relationship between cigarette sales in the U.S. as a proxy for use and percent of U.S. adults who currently smoke. Predictions from a linear regression are plotted (dark line) with upper and lower 95% confidence intervals (shaded area). Points represent data from the CDC’s Behavioral Risk Factor Surveillance System survey for smoking prevalence and U.S. Alcohol and Tobacco Tax and Trade Bureau for cigarette sales for the year labeled in the plot.

The propagated error for total cigarette butt litter and area-weighted mean density were 0.1% and 0.07% of the average, respectively. The total number of cigarette butts littered per year estimated from the model was within 2.5% of the mean 2018–2020 TTB estimate (145.77 billion vs 142.18 billion from TTB, assuming 65% of consumed cigarettes were littered; Fig 3). National cigarette sales in the U.S. were significantly correlated to smoking prevalence measured in the BRFSS over the period from 1995–2021 (*t-value*_*1*,*25*_ = 11.9, *r*^*2*^ = 0.84, *p* <0.0001; Fig 3). Using these trend results, the model predicts a >50% decrease in annual littering in 2018–2020 compared to 1995.

The effect of varying littering rates from the Schultz et al. estimate of 65% to 85%, 35%, and 10% of cigarettes resulted in a range of national, area-weighted average cigarette butt litter density of 0.024, 0.010, and 0.003 butts/m^2^, respectively. The magnitude of cigarette butt littering varied among the littering scenarios, however, the spatial patterns and differences among quintiles of SER remained constant (Table 4).

**Table 4:** Comparison of littering behavior scenarios.

	Schultz et al. 2013 (65%)		85%		35%		10%	
SER quintile	Butts per m^2^	IRR	Butts per m^2^	IRR	Butts per m^2^	IRR	Butts per m^2^	IRR
0.0–0.2	0.0153		0.0200		0.0083		0.0024	
0.2–0.4	0.0112	0.73	0.0146	0.73	0.0060	0.73	0.0017	0.73
0.4–0.6	0.0135	0.88	0.0177	0.88	0.0073	0.88	0.0021	0.88
0.6–0.8	0.0274	1.79	0.0359	1.79	0.0148	1.79	0.0042	1.79
0.8–1.0	0.0858	5.60	0.1122	5.60	0.0462	5.60	0.0132	5.60
**Total cigarette butts**	**145,772,501,112**		**190,625,578,336**		**78,492,885,197**		**22,426,538,628**	

Data represent the area-weighted littered cigarette butt density (butts/m^2^) among quintiles of Social-Environmental Risk (SER) and incidence rate ratios (IRR) for the Schutlz et al 2013 scenario (65%) compared to littering of 85%, 35%, and 10% of all cigarettes. Incidence rate ratios and intervals are back-transformed from the log scale. The total modeled number of cigarette butts littered in a year is presented for each scenario.

## Discussion

We conducted a national-scale environmental justice assessment of cigarette butt litter estimated with a place-based model using publicly available data for the contiguous United States. The model estimates from this study represent the first national, spatially-explicit estimate of cigarette consumption and littering at the census tract scale. The estimate allows a cross-scale assessment of potential cumulative impacts from neighborhoods to the nation and advances our understanding of the spatial scale and magnitude of the cigarette butt litter problem in the U.S. We found that littered cigarette butts are distributed heterogeneously, and census tracts with high modeled cigarette butt litter were clustered around urban areas particularly in neighborhoods already facing high environmental burdens and social vulnerabilities. The disproportionate impacts on these communities from the disposal phase of the cigarette lifecycle is an environmental justice issue.

Although cigarette butt litter has long been recognized as a major environmental cost associated with tobacco use, the available information about cigarette butt littering is limited to trash clean ups at specific locations and relatively few published studies. Cigarette butt litter introduces toxic chemicals and microplastics into the environment, potentially affecting human and environmental health for years after introduction and imposing a considerable financial burden on communities exposed to and tasked with cleaning up this waste. Our results showed cigarette butt densities ranged as high as 45.5 butts/m^2^ and were significantly higher in metropolitan areas and census tracts experiencing the greatest SER. The model shows that the distribution and magnitude of cigarette use and butt litter disproportionately affected communities that already suffer SER due to a suite of intersectional factors that have led to environmental and health inequalities [1]. The higher rates of use in areas with elevated SER mirror studies documenting higher cigarette consumption [28, 29] and greater exposure to secondhand smoke in communities of low SES and communities of color [97]. While the estimates of littered cigarettes butts represent a single year, their potential health effects may last for years via accumulated cigarette butt litter. The results also provide a key resource for addressing cumulative impacts affecting U.S. communities that can be integrated with existing datasets and tools at the localized scales necessary for addressing health disparities [98, 99].

Our results advance our understanding of where cigarette butt litter enters the environment, providing the basis for policy at local, state, and federal levels. The significant relationships among cigarette butt density and SER are not meant to infer causality, but rather to highlight the co-occurrence of tobacco-related environmental hazards and other existing environmental and socioeconomic determinants of health. High density metropolitan and micropolitan areas (Figs 1 and 2) may face the worst effects of cigarette butt litter, despite higher smoking prevalence in rural areas [90]. This difference can be explained by the significant effect of population density on census tract cigarette butt litter (Fig 2). While modeled cigarette butt density was significantly higher in the top quintiles of SER, the relationship to population density explains the orders of magnitude range of cigarette butt density among census tracts within a quintile (Fig 2). Small, high population density census tracts with existing high SER are most likely to be impacted by cigarette butt litter as high as 45.5 butts/m^2^. This is a critical result that helps identify the most impacted areas and direct research, education, and policy interventions to document and curtail disproportionate environmental impacts from cigarette waste.

The model provides place-based estimates of cigarette butt litter at locations across the U.S. using publicly available data. It is important to consider these results in the context of patterns of cigarette usage and the limitations of the available data. The model uses data from different sources and years: census data from 2020, consumption data from 2018–2019, smoking prevalence data from 2020, and littering estimates from 2013. Estimates derived from self-reported surveys may contain bias such as nonresponse or measurement bias [100, 101]. Furthermore, our smoking prevalence and CPD estimates were taken from different surveys, with their own measurement and methodological approaches, including the geographical level at which estimates were generated. The BRFSS smoking prevalence data excludes individuals who occasionally smoke, and the TUS-CPS estimates of daily cigarette consumption are ~20–50% lower than other published estimates (Table 1), both of which may bias the model results low. Total U.S. cigarette consumption decreased from 301 billion cigarettes in 2010 to 216 billion in 2020, down from nearly 500 billion in 1995 (Fig 3). The number of cigarette butts collected along U.S. roads and waterways decreased between 2009–2020, yet still amounted to ~9.7 billion cigarette butts collected by one organization in the U.S. in 2020 [102]. This result has limited value for extrapolating to total littered cigarettes beyond the fact that, at a minimum, collected cigarette butts equated to ~4.3% of cigarettes consumed in 2020 (Fig 3). The lack of standardized studies prevents statistical analysis of this trend or direct comparison of the number of cigarettes collected during cleanups to model results. Given that cigarette butts can persist in the environment for more than a decade and continue to have negative environmental impacts throughout their lifespan [58, 103, 104], the current estimates may also underestimate the cumulative effects of cigarette butt litter. As such, the model output represents the low end of cigarette butts affecting U.S. census tracts and, while the patterns are robust, the cigarette butt densities for specific census tracts should be treated as relative estimates. Regardless, the results align with independent measures of total cigarette consumption (Fig 3), estimates of cigarette butt densities in the literature (mean = 0.001–2.7 butts/m^2^, max = 48.8 butts/m^2^) [39, 45, 59], and the BRFSS smoking prevalence measure used in the model tracks trends in overall consumption over time (Fig 3), providing strong evidence that the model accurately reflects cigarette use in the U.S.

The modeled cigarette butt density was sensitive to the estimate of the percent of cigarettes that are littered, with the output being directly proportional to this value. The benefit of this model structure is that it is simple to adapt to improved estimates of littering behavior. The lower estimate of 10% littering rate, which is lower than many other values reported in the literature, still resulted in considerable amounts of cigarette butts entering the environment. Importantly, changes to the estimate of the percent of cigarettes littered did not change the spatial patterns that raise an environmental justice concern (Table 4). The lack of population dependent or spatially-explicit littering rates required us to assume a universal rate of littering. This is unlikely, as both individual characteristics and environmental context are important factors determining littering behavior [37]. The model attributes littered butts to where people live, yet littering is often concentrated outside of places of work, schools, restaurants, or bars that may be in a different census tract than a person’s residence [63, 105, 106]. This bias may be most evident in the ~15% of census tracts designated as RUCA “commuter zones” where a considerable fraction of residents commute to other census tracts for work (Table 2); commuting likely concentrates litter along roadways and near workplaces in urban areas. Additionally, the littering estimates (Table 1) tend to rely on behavioral observations in public settings where cigarette consumption and littering rates may be quite different from private settings (e.g., at home), but these patterns have not been documented. Nevertheless, spatially-explicit littering rates are unlikely to change the patterns and correlations observed here since much of the spatial pattern is overwhelmingly driven by smoking prevalence and population density (Fig 2). Moreover, the factors contributing to demographic or spatially-varying littering behavior may worsen the observed disparities because of the correlation among social factors and environmental inequities; age, gender, income, presence of litter, and availability of trash receptacles all influence littering rates [37, 81, 82]. Furthermore, the cost of cleanup may prevent removal in some areas and create a feedback loop that encourages littering in localities lacking funds for neighborhood cleanups [37].

The present findings are consistent with evidence that historically marginalized communities disproportionately bear the burden of tobacco-related morbidity, mortality, and environmental degradation. Previous studies have identified disproportionate effects of marketing and use of tobacco products on marginalized communities [21, 23, 28, 107]. Here, we add that cigarette butt litter is a nationwide issue in the U.S., with the greatest amounts of litter falling on neighborhoods located in urban areas that already have the highest exposure to environmental burdens and social vulnerabilities. This finding is critical to efforts aimed at addressing environmental justice and health inequity related to tobacco products [108]. The power of this model is the ability to scale from neighborhoods to the nation. Future research may explore spatial and demographic-specific littering behavior. While national cigarette consumption and smoking prevalence have each fallen ~30% over the lifetime of a cigarette butt (Fig 3), the interacting effects of population change, smoking prevalence, and behavior likely lead to local patterns diverging from the national trend. The small-scale estimates could support local efforts at managing cigarette litter. Two important determinants of littering behavior are the beliefs that butts are litter and that seeing litter is a nuisance [82, 109]. Targeted education programs that bring attention to the toxic and long-lasting impacts of cigarette butts can be effective at changing beliefs about cigarette butt litter in areas with the highest litter [80, 83]. At the federal level, recent Executive Order 14096 [65] and the White House Council on Environmental Quality revisions to NEPA [110] refocus environmental justice as a priority throughout the federal government including NEPA implementation. Our results documenting environmental justice impacts of cigarette butt litter can inform the evaluation of indirect and cumulative environmental impacts of disposal of cigarettes and other tobacco products as part of NEPA-mandated evaluations [69]. Our results can also help address considerations of related actions and communities with environmental justice concerns in multiple local, regional, and national contexts [110]. The finding that the combination of smoking prevalence and population density determined nationwide patterns of cigarette butt density and correlated to decreased cigarette consumption over the last 25 years highlights the fact that reducing smoking rates is a key solution to this issue that would simultaneously reduce other associated human and environmental health hazards.

## Supporting information

S1 Data Source data and model output. (XLSX)
